# Prognostic significance and gene co-expression network of CD16A and FGL2 in gliomas

**DOI:** 10.3389/fonc.2024.1447113

**Published:** 2024-11-19

**Authors:** Ziwen Cao, Xing Liu, Jun Yan

**Affiliations:** ^1^ Beijing Institute of Brain Disorders, Laboratory of Brain Disorders, Ministry of Science and Technology, Collaborative Innovation Center for Brain Disorders, Capital Medical University, Beijing, China; ^2^ Department of Neuropathology, Beijing Neurosurgical Institute, Beijing, China

**Keywords:** FCGR3A, FGL2, glioma, prognosis, gene network

## Abstract

**Introduction:**

The CD16A protein encoding gene FcγRIIIa (*FCGR3A*) and its potential ligand Fibrinogen-like protein 2 (*FGL2*) are involved in various cell physiological activities on the extracellular surface. Aberrant expression of these genes has been linked to tumorigenesis.

**Methods:**

To assess the prognostic significance of *FCGR3A* and *FGL2* transcription expression in glioma and explore their roles in glioma initiation and progression, we utilized multiple online databases, including TCGA, GEPIA, CGGA, cBioPortal, TISCH, LinkedOmics, Ivy Glioblastoma Atlas Project, and Human Protein Atlas.

**Results:**

Our analysis revealed that *FCGR3A* and *FGL2* expression was significantly correlated with clinical variables such as age, tumor type, WHO grade, histology, IDH-1 mutation, and 1p19q status. A strong correlation was also observed between the transcriptional expression levels of *FCGR3A* and *FGL2*. High expression of both genes predicted poor prognosis in primary and recurrent glioma patients, particularly those with lower grade gliomas. Cox regression analysis further confirmed that elevated expression of *FCGR3A* and *FGL2* were independent prognostic factors for shorter overall survival in glioma patients. Gene co-expression network analysis suggested that *FCGR3A*, *FGL2*, and their co-expressed genes were involved in inflammatory activities and tumor-related signaling pathways. Additionally, tissue microarrays from glioma patients at Tiantan Hospital showed significantly higher *FCGR3A* protein expression in high-grade gliomas compared to low-grade gliomas.

**Discussion:**

In conclusion, our findings suggest that *FCGR3A* and *FGL2* could serve as promising prognostic biomarkers and potential therapeutic targets for glioma patients.

## Introduction

Glioma, a prevalent category of brain tumors with a high mortality rate, represents the predominant form of primary malignant brain tumors in adults, albeit accounting for less than 1% of all newly diagnosed tumors (1). Among various diffuse gliomas, glioblastoma (GBM) comprises 70-75% of cases and carries the gravest prognosis, with a median survival rate of under 2 years even after standard chemoradiotherapy (2). Molecular therapies targeting epigenetic modifications are currently under investigation and are anticipated to usher in a significant advancement in treating malignancies such as GBM (3). Mounting evidence indicates that an integrated histological-molecular classification system might surpass the conventional histological approach. Biomarkers like isocitrate dehydrogenase 1 (IDH-1) and O6-methylguanine-DNA methyltransferase (MGMT) are pivotal not just for diagnosis and prognosis but also as potential therapeutic targets (4, 5). Delving deeper into these biomarkers lays a crucial foundation for tailored treatments against specific glioma subtypes.

FCGR3A, also known as CD16A, encodes a receptor for the Fc portion in IgG antibodies, which plays a crucial role in immune responses, especially in the phagocytosis of immune complexes and antibody-dependent cellular cytotoxicity (ADCC) (6–8). FGL2 encodes a fibrinogen-like protein that has been implicated in various immune and coagulation processes, including the regulation of T-cell responses and the promotion of thrombosis (9, 10). In recent years, increasing evidence has linked the FCGR3A or FGL2 effect to both chronic and malignant diseases. For instance, abnormal expression or dysfunction of FCGR3A has been associated with autoimmune diseases like rheumatoid arthritis and immune thrombocytopenic purpura, as well as malignancies such as leukemia and lymphomas (11–13). Similarly, FGL2 has been found to be overexpressed in certain cancers, including breast cancer, colon cancer, and melanoma, where it contributes to tumor growth and metastasis (14–18). Additionally, FGL2 are involved in complex tumor invasion and cell migration processes, which can lead to more aggressive tumor phenotypes and poorer patient prognosis (19). Ongoing research continues to explore the intricate roles of FCGR3A and FGL2 in health and disease, aiming to develop new therapeutic strategies that target this system to improve patient outcomes.

FGL2 has been shown to function as a promoter of glioblastoma progression and of stem-like transition of glioma cells by augmenting immunosuppression. FcγRIIB and FcγRIII are both the receptors for FGL2. The binding of FGL2 to FcγRIIB, a suppressive FcγR, has been shown to results in B/T cell apoptosis and inhibition of DCs maturation, play an important role in FGL2-induced immunosuppression. FcγRIII, also known as CD16, is expressed on many leukocytes and works as a marker for a subset of human monocytes. However, binding of FGL2 to FcγRIII on DC but lacking FcγRIIB had no apparent effect. We previously found that FGL2 produced by glioma cells acts on the CD16 receptor of macrophages in the microenvironment through paracrine signaling. Through the CD16/SyK/PI3K/HIF1α pathway, it recruits and differentiates macrophages, inducing macrophage differentiation into CXCL7^+^ macrophages (20, 21). Despite the growing interest in FCGR3A and FGL2 effect in glioma, their clinicopathological significance and prognostic value have received less attention. There is a need for more comprehensive and updated analyses to confirm and expand upon these initial observations. This study could provide deeper insights into the prognostic value of FCGR3A and FGL2 expression in glioma, potentially leading to improved patient stratification and treatment strategies.

Different kinds of online databases, tools and integrate data were applied in this study. First, the transcription expression level of FCGR3A/FGL2 among glioma patients were investigated. Then, their relations with clinical parameters were analyzed, while the prognostic factors were also analyzed. Furthermore, potential gene functions and pathways were predicted through data mining.

## Materials and methods

### Ethics statement

This study was approved by the Academic Committee of Capital Medical University, and conducted according to the principles expressed in the Declaration of Helsinki. All the datasets were retrieved from the published literature, so it was confirmed that all written informed consent was obtained.

### GEPIA database

GEPIA (Gene Expression Profiling Interactive Analysis) (http://gepia.cancer-pku.cn) is a recently launched web-based interactive tool designed for the analysis of RNA sequencing and expression data. It draws from a vast dataset comprising 9736 tumor samples and 8587 normal samples sourced from The Cancer Genome Atlas (TCGA) and Genotype-Tissue Expression (GTEx) projects, employing a standardized data processing approach. In our study, we leveraged GEPIA to compare transcriptional expressions between gliomas and healthy brain tissues. Additionally, we performed a survival analysis among glioma patients categorized by different WHO grades.

### CGGA database

The Chinese Glioma Genome Atlas (CGGA) database (http://www.cgga.org.cn) serves as an online repository and analytical tool, specializing in brain tumor datasets from over 2,000 Chinese patients. This comprehensive database encompasses whole-exome sequencing, DNA methylation profiles, mRNA sequencing, mRNA microarray data, microRNA microarray data, along with corresponding clinical information. Clinicopathological data was sourced from the CGGA database. We conducted a survival analysis on a selected cohort of glioma patients and further performed a Pearson correlation analysis to assess relationships within the dataset.

### cBioPortal

The cBio Cancer Genomics Portal (cBioPortal) (https://www.cbioportal.org) is an online platform designed for exploring, visualizing, and analyzing complex cancer genomics data. It integrates various genomic data types, such as somatic mutations, DNA copy number variations, mRNA and microRNA expression levels, DNA methylation patterns, protein abundance, and phosphoprotein levels. We utilized the cBioPortal to assess the frequency of gene alterations and conducted a survival analysis comparing patients with and without specific gene alterations.

### LinkedOmics

LinkedOmics (http://www.linkedomics.org) is a publicly accessible web-based platform housing multi-omics data across all 32 TCGA cancer types. It comprises three analytical modules: LinkFinder, LinkInterpreter, and LinkCompare. Using LinkFinder, one can identify attributes related to a specific query, such as mRNA or protein expression signatures linked to genomic alterations, potential biomarkers for clinical features, and target genes of transcriptional factors, microRNAs, or protein kinases. To derive biological insights from these associations, the LinkInterpreter module performs an enrichment analysis leveraging Gene Ontology, biological pathways, network modules, and other functional categories. In our study, we employed both LinkFinder and LinkInterpreter to investigate the underlying gene regulatory network.

### TISCH

The Tumor Immune Single Cell Hub (TISCH) is a resource of single-cell RNA-seq (scRNA-seq) data from human and mouse tumors, which enables comprehensive characterization of gene expression in the tumor microenvironment (TME) across multiple cancer types. Overall, the TISCH database contains 76 high-quality tumor datasets across 27 cancer types and 3 PBMC datasets.

### Ivy Glioblastoma Atlas Project

Gene expression in the various anatomical regions of glioblastoma tumors was analyzed using the Ivy GAP (http://glioblastoma.alleninstitute.org/). For RNA sequencing and analysis, raw gene-level values of fragments per kilobase of transcript per million reads mapped (FPKM) and the associated clinical data were acquired from the publicly available Ivy Glioblastoma Atlas Project database. Normalized Ivy Glioblastoma Atlas Project were downloaded from GlioVis (http://gliovis.bioinfo.cnio.es/).

### Human Protein Atlas

Human Protein Atlas (HPA, https://www.proteinatlas.org/) is a human proteome database based on quantitative transcriptomic analyses on tissue and organ levels. We collected tissue-specific protein expression data, confocal images and annotations of the protein subcellular distribution from version 7.0 of HPA database.

### RNA-seq data and processing

The RNA-seq expression data of TCGA cohort and CGGA trial were retrieved along with the process of acquiring clinical information and were transformed as log2 (FPKM+1) for downstream analysis. Based on TCGA data, CIBERSORT was constructed to calculate the relative proportion of 22 immune cell types.

### Tissue microarray

Clinical prognosis of FCGR3A/FGL2 was confirmed by histology and immunohistochemistry. The production of tissue microarrays involves Standardized sample collection, formalin fixation, array fabrication, and slicing. Subsequently, the tissue microarrays undergo standard histological staining procedures, often involving hematoxylin and eosin (H&E) staining, which highlights cellular structures and provides contrast for detailed histological examination. Additionally, immunohistochemistry (IHC) staining of CD16A and FGL2 performed to detect specific antigens in the tissues, further enhancing the diagnostic and prognostic value. Once stained, a panoramic scan of the entire tissue microarray was performed. CD16A is localized to the cell membrane, while FGL2 can be localized to the cytoplasm, cell membrane, and intercellular space.

### Cell culture and immunofluorescent staining

THP-1 (human monocytic leukemia) is derived from the blood of a patient with acute monocytic leukemia, and in experimental studies, it is often induced to differentiate into monocyte-derived macrophages. THP-1 cells are induced to differentiate into macrophages (M0) using phorbol ester (PMA), and then cultured for 48 hours in the presence of LPS (100ng/mL) and IFN-γ (20ng/mL) to induce their polarization toward M1, or with IL-4 (20ng/mL) and IL-13 (20ng/mL) to induce their polarization toward M2, all while PMA is still present. After polarization, the cells are resuspended in serum-free RPMI-1640 medium without stimulants and PMA, and harvested 24 hours later.

Discard the culture medium and slowly add PBS at room temperature to the Petri dish to remove suspended cells. Cover the cells with 4% neutral formaldehyde fixing solution, place them at 4°C, and fix them for 15 minutes. After washing the fixing solution, block the non-specific binding sites with 5% blank goat serum for 30 minutes at room temperature. Remove the blocking solution, add diluted primary antibody, and incubate at 4°C overnight. The next day, incubate the secondary antibody in the dark at room temperature for 1 hour. Add DAPI working solution to the sample, incubate in the dark at room temperature for 10 minutes. After adding anti-fluorescence attenuation sealing agent, observe and collect images under a fluorescence microscope.

### Antibody information

FGL2 -Novus-H00010875-M01 (mouse react to human)
FGL2 -Sigma-Aldrich-HPA021011-100UL (rabbit react to human) CD16-Abcam-ab246222 (rabbit react to human)
CD32- Abcam-ab282740 (mouse react to human)

### Recombinant protein

PMA-MCE-16561-29-8
LPS-MCE-HY-D1056
IFN-γ-MCE-HY-P70610G (Species-human)
IL-4-MCE- HY-P78549 (Species-human)
IL-13- Abcam-ab270079 (Species-human)

### Statistical analysis

Kruskal-Wallis test was used to depict the diversity of immune infiltration in FGL2-FCGR3A-stratified groups. Kaplan-Meier method was used to assess 5-year OS and RFS, whereas logrank test and Cox regression models were applied for the assessment of the prognostic and risk significance. Spearman correlation analysis was performed to investigate the bivariate correlation. The χ2 test was applied to depict the relation of clinicopathological parameters, molecular subtypes. The two-sidedp ≤ 0.05 was considered statistically significant. The data were analyzed through IBM SPSS Statistics V.20.0 and R software V.4.0.4.

## Results

### Association of FCGR3A and FGL2 expression with disease progression in glioma

**Figure 1:**
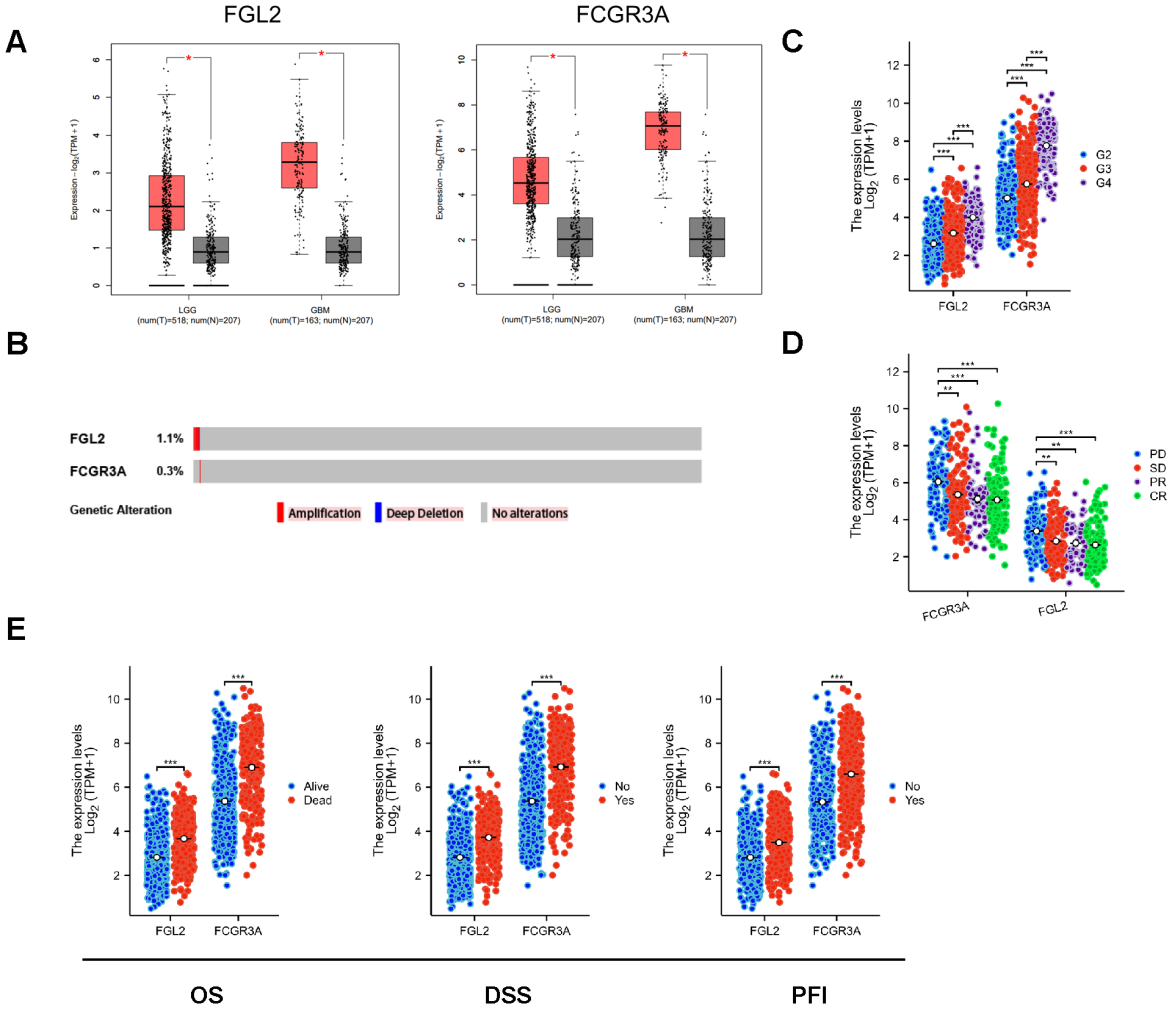
Transcriptional expression of FCGR3A and FGL2. **(A)** Transcriptional expression of FCGR3A and FGL2 in gliomas and normal brain tissues(GEPIA). **(B)** Genetic alterations of FCGR3A and FGL2 in gliomas (cBioportal). **(C)** Transcriptional expression of FCGR3A and FGL2 in each grade of gliomas (GTEx). **(D)** Transcriptional expression of FCGR3A and FGL2 in relative treatment outcomes (TCGA). **(E)** Transcriptional expression of FCGR3A and FGL2 in multiple survival times (TCGA). *p < 0.01, **p < 0.001, ***p < 0.0001.

To investigate whether there were differences between glioma and normal brain tissue, the gene expression data of the FCGR3A and FGL2 were obtained from TCGA and GTEx database in. As shown in **Figure 1A**, mRNA expressions of FCGR3A and FGL2 were significantly upregulated in Low-grade gliomas and glioblastomas respectively compared to normal brain tissue, and there are few mutations in either 2 genes (**Figure 1B**). Therefore, the upregulation of FCGR3A and FGL2 expression is induced by the tumor. The mass spectrometry results show that the abundance of FCGR3A and FGL2 proteins were also upregulated in glioma tissues in the CPTAC database (**Supplementary Figures 1A–D**). The transcription and translation of genes are influenced by epigenetics and post transcriptional modifications. According to the results of the TCGA methylation chip, the methylation levels of the FCGR3A and FGL2 promoter regions are down regulated, and mRNA upregulation may be related to this (**Supplementary Figures 1E, F**). FCGR3A is regulated by the GATA family and pro-inflammatory transcription factors such as STAT1 and NFKB1, while FGL2 is targeted and inhibited by many miRNAs (**Supplementary Figures 1G, H**). According to the sequencing results of normal tissues in the GTEx database, FCGR3A is highly expressed in peripheral blood and spleen, and partially expressed in the lungs. FGL2 is highly expressed in the esophagus and arteries. The expression level of these two genes was very low or hard detectable in 13 brain regions (**Supplementary Figure 2A**). It shows that the high expression of FCGR3A and FGL2 in glioma is induced by tumor cells or immune cells infiltrated into the brain. Pan cancer analysis confirms this hypothesis that changed expression of FCGR3A and FGL2 occurs in most tumor types (**Supplementary Figures 2B, C**). The increased expression of FCGR3A and FGL2 is associated with various clinical features in glioma. FCGR3A and FGL2 are highly expressed in elderly individuals (**Supplementary Figure 2D**), glioblastoma patients (G4) (**Figure 1C**), those with tumor progression after initial treatment (**Figure 1D**), and those with poor prognosis confirmed by OS, DSS and PDI (**Figure 1E**). IDH mutations and prognostic features such as 1p9q co del are negatively correlated with the expression levels of FCGR3A and FGL2 (**Supplementary Figures 2F, G**). It’s worth noting that, excluding GBM samples, the histopathological types of LGG patients are also related to FCGR3A and FGL2. Astrocytoma has the highest gene expression, oligodendroglioma has the lowest, and the mixed type is between the two (**Supplementary Figure 2H**).

### Association of mRNA expression of FCGR3A and FGL2 in glioma patients

**Figure 2:**
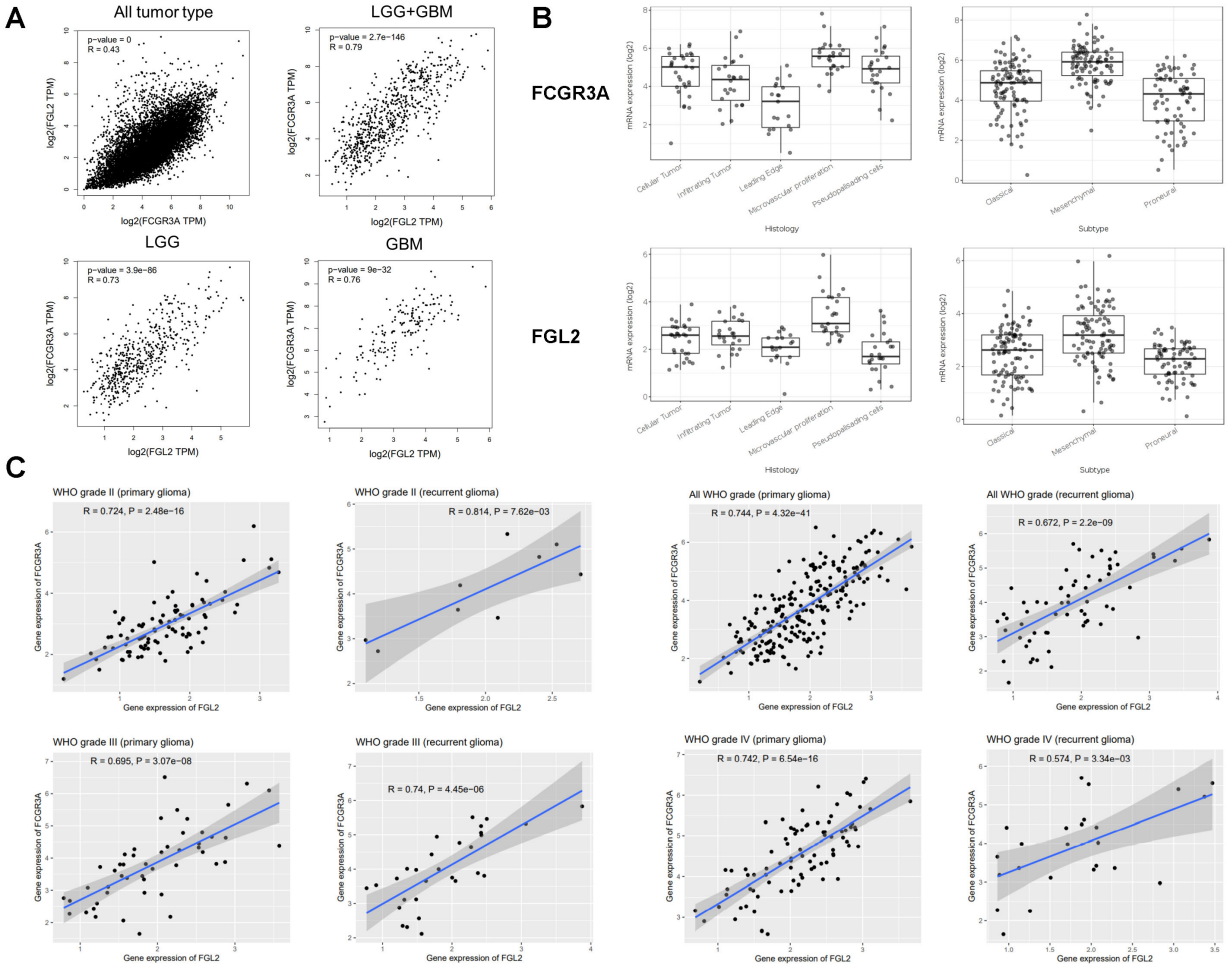
Genetic correlation of FCGR3A and FGL2 with Pearson correlation analysis. **(A)** Linkedomics. **(B)** Iyy GAP database. **(C)** CGGA database.

As shown in **Figure 2B**, the anatomical localization of FCGR3A and FGL2 within the tumor is the same, both highly expressed in the cellular tumor region associated with differentiation and growth (Cellular Tumor, CT) and angiogenesis region associated with angiogenesis, immune regulation, and response to wounding (Microvascular Proliferation, MVP). Among the three transcriptional subtypes of glioma, the mesenchymal subtype tumors have the highest expression level of FCGR3A and FGL2 as compared with classical or proneural subtypes (**Figure 2C**). These data indicate that the expression level of FGL2 and FCGR3A is associated with high cell growth, angiogenesis, immune regulation, and malignant potential. Based on these data, we can speculate that cells co expressed with FCGR3A and FGL2 migrate from peripheral blood into the tumor.

Given that the expression levels of FCGR3A and FGL2 exhibit similar trends across different subtypes of gliomas and the anatomical localization is the same in tumor, we propose that FCGR3A and FGL2 co express in gliomas. As shown in **Figure 2A**, FCGR3A is significantly positively correlated with FGL2 expression in all tumor types (*R*=0.43), while the correlation between FCGR3A and FGL2 is more prominent in glioma samples (*R*=0.79). The above TCGA results can be validated by the CGGA database. The correlation between FCGR3A and FGL2 is not affected by race, grade, primary or recurrent factors, and remains highly positively correlated in Asian populations (**Figure 2C**).

### Prognostic value of FCGR3A and FGL2 transcription expression in glioma

**Figure 3:**
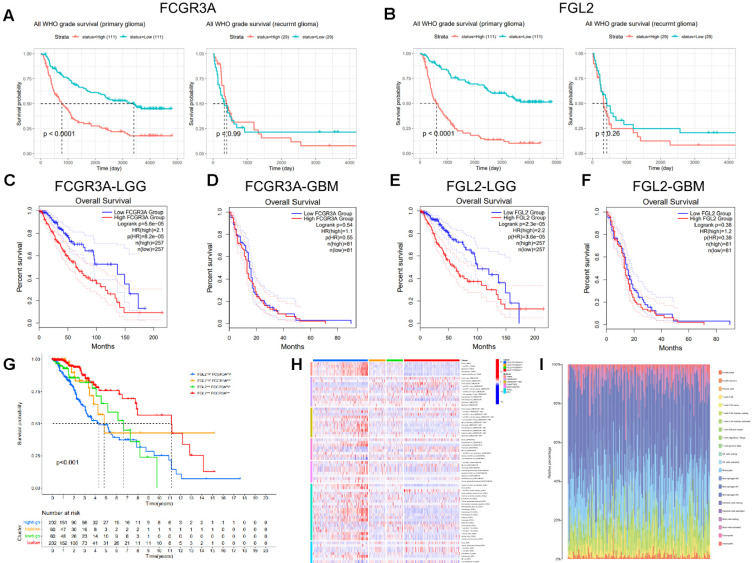
Transcriptional expression of FCGR3A and FGL2 association with OS in gliomas. **(A, B)** CGGA database. **(C-F)** TCGA database. Divide TCGA glioma samples into four quadrants, compare the OS **(G)** and immune infiltration **(H, I)**.

Given the location and the clinical characteristic of tumors with high expression of FGL2 and FCGR3A prognostic value were evaluated. In the CGGA database, high expression of FCGR3A or FGL2 in glioma was associated with a shorter OS in primary tumor, whereas no difference was found between high and low expression in recurrent glioma (**Figures 3A, B**). In the GEPIA database (**Figures 3C–F**), high expression of FCGR3A or FGL2 indicated poor prognosis in WHO grade II and III patients. Similarly, the overall survival was unchanged in WHO grade IV. To further determine the prognostic role of co-expression, TCGA samples were grouped into four clusters according to the median expression of FCGR3A and FGL2, and found that patients with low expression of both FCGR3A and FGL2 had the longest survival time (**Figure 3G**). The prognostic indicators of FCGR3A and FGL2 in the glioma subgroup can be seen in **Supplementary Figures 3**, **4**. In summary, an effective overall survival prognosis model can be constructed by utilizing the expression levels of FCGR3A and FGL2, with AUC areas higher than 0.7 within 10 years (**Supplementary Figure 5**). In addition, co expression of FCGR3A and FGL2 significantly regulates immune infiltration. The co-high expression group had significantly higher immune cell scores, especially macrophage scores according to the immune infiltration with multiple algorithms in the same grouping (**Figures 3H, I**). Therefore, the poor prognosis of FGL2^hi^FCGR3A^hi^ group patients could be induced by the robust immune infiltration and inflammation in the tumor.

### Gene set enrichment analysis of FCGR3A and FGL2 functional networks in glioma

**Figure 4:**
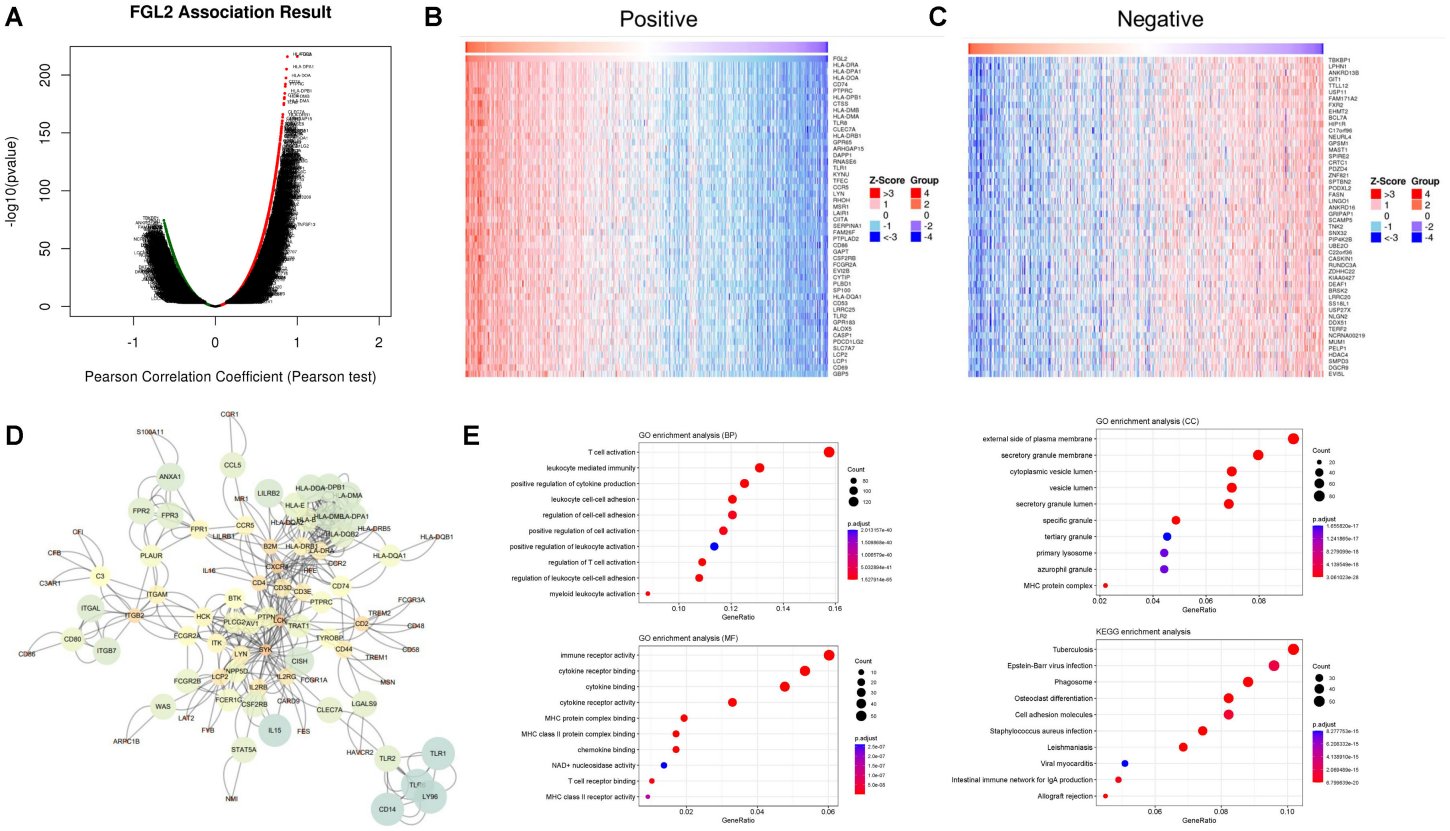
FGL2 related pathways in gliomas. **(A–C)** Co-expression genes in gliomas (LinkedOmics). **(D)** PPI of co-expression genes. **(E)** GO enrichment of co-expression genes.

**Figure 5:**
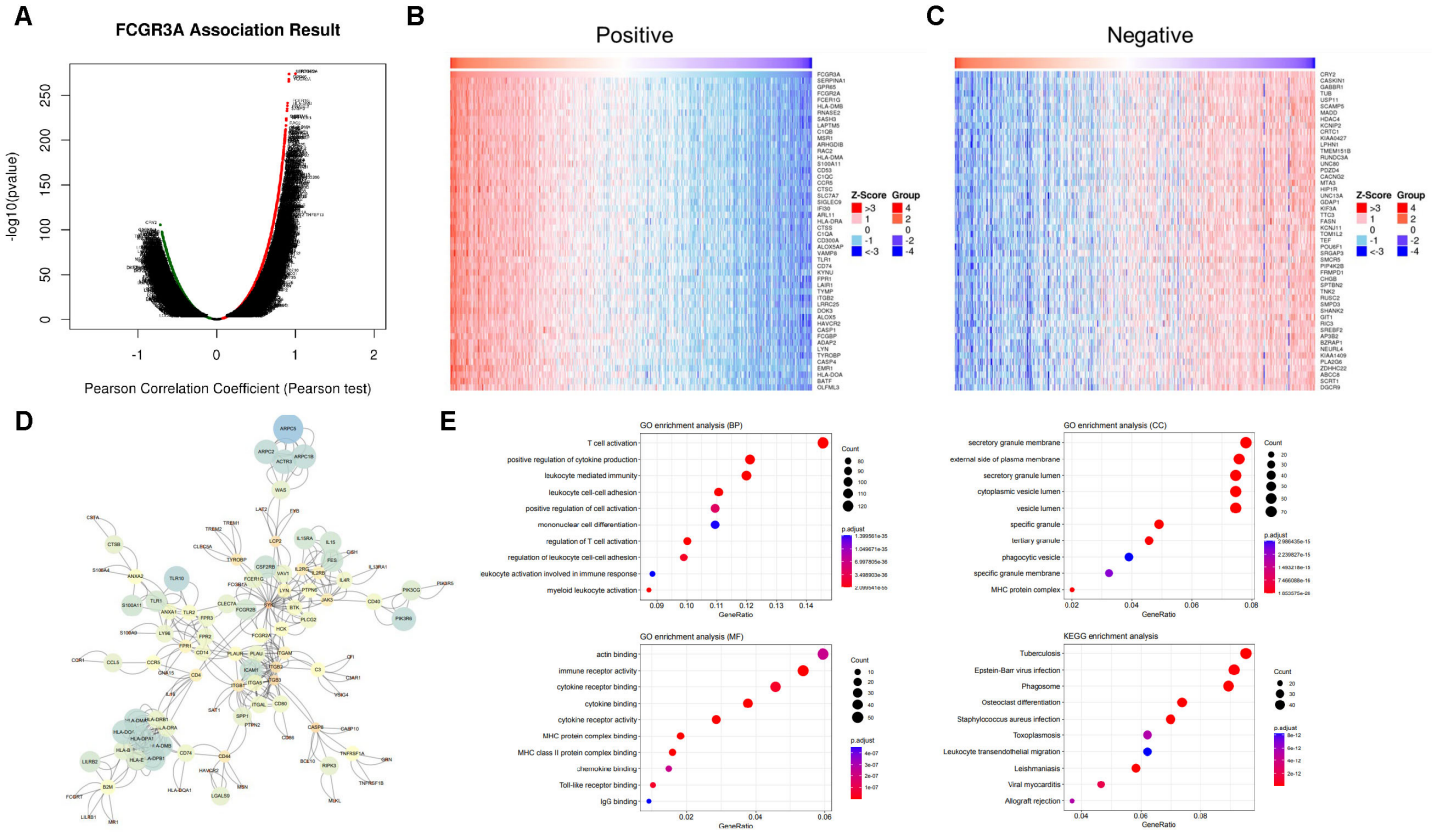
FCGR3A related pathways in gliomas. **(A–C)** Co-expression genes in gliomas (LinkedOmics). **(D)** PPI of co-expression genes. **(E)** GO enrichment of co-expression genes.

Genes that are highly positively and negatively correlated with FGL2 were screened for pathway enrichment and protein-protein interaction analysis (**Figure 4**). The results showed that top 50 genes with high positive correlation in FGL2 contained many HLA molecules, such as HLA-DRA, HLA-DPA, HLA-DOA, HLA-DPB, HLA-DNM, HLA-DMA, HLA-DRB, CD74 and CD86 genes, suggesting that the function of FGL2 was highly synergistic with antigen presentation. On the other hand, FGL2 was positively correlated with a variety of pattern recognition receptors, including TLR8, TLR1, and TLR2, in which TLR1 and TLR2 were in the cell membrane, and TLR8 was in the endosome, and the former two genes assisted monocytes to phagocytose antigens, the antigens entered the cell with the assistance of TLR8 to induce the production of IFN-α (**Figures 4A–C**). Therefore, FGL2 is associated with T cell activation, immune cell migration, cytokine secretion, and MHCII complex formation pathways in pathway enrichment (**Figures 4E**). The results of PPI showed that the FGL2-related genes were centered on LCK and SYK, which both belonged to the downstream regulators of ITAM and were related to immune cell signal transduction and activation (**Figure 4D**). On the other hand, genes that are highly positively related to FCGR3A include FCGR2A, FCER1G, and FCGBP, which belong to the FC receptor family too, and a variety of complement-related genes have also been screened, such as C1QB and C1QC (**Figures 5A–C**). In terms of pathway enrichment, the functions of FCGR3A and FGL2 completely overlapped, indicating that their functions are synergistic and complementary, mainly responsible for recruiting and activating T cells (**Figure 5E**). The results of protein-protein interaction showed that SYK was also the core of FCGR3A-related genes (**Figure 5D**). Therefore, SYK is the key element for the downstream kinases of FCGR3A and FGL2.

**Figure 6:**
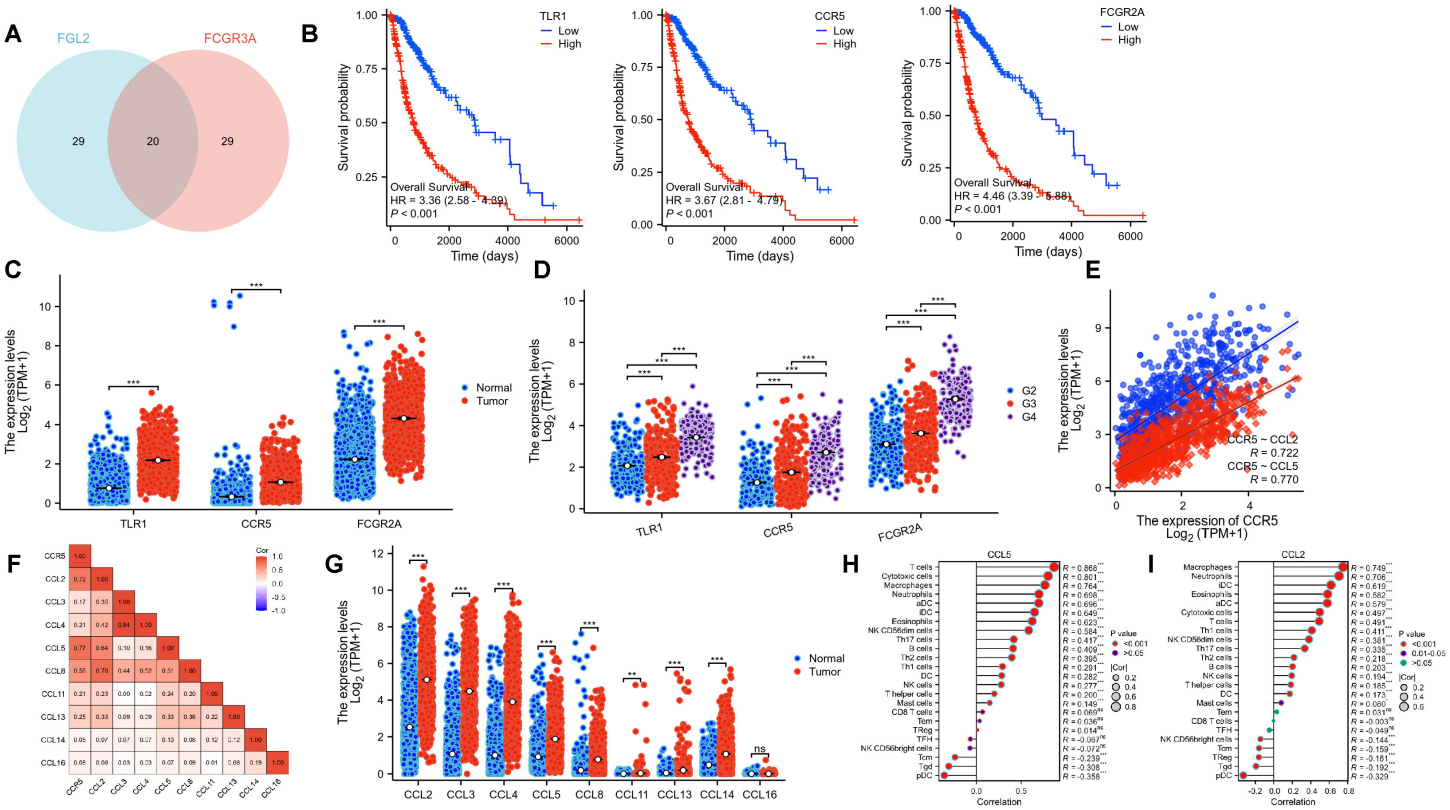
FCGR3A and FGL2 related pathways in gliomas. **(A)** The intersection of the top 50 positively correlated genes. **(B)** KM curves of three hub genes. **(C, D)** Transcriptional expression of three hub genes in gliomas and normal brain tissues. **(E, F)** Genetic correlation of CCR5 and its ligands. **(G)** Transcriptional expression of CCR5 ligands. **(H, I)** Immune infiltration of CCL5 and CCL2. *p < 0.01, **p < 0.001, ***p < 0.0001, ns: P > 0.05.

Since the previous analysis confirmed that the prognostic regulation of FCGR3A and FGL2 is related to immunity, we further explored the mechanism by taking the intersection of top50 positive related genes. **Figure 6** shows that among the 20 intersecting genes, there are 3 immune related molecules, TLR1, CCR5, and FCGR2A. These three genes are significantly associated with poor prognosis and are highly expressed in tumor samples, and their expression increases with malignancy. It is worth noting that CCR5 is highly positively correlated with both genes and has been reported to be associated with neurological inflammation, which may be the key to the recruitment of CD16+ monocytes into the brain.

### Biological role of FCGR3A and FGL2 double-positive monocytes

**Figure 7:**
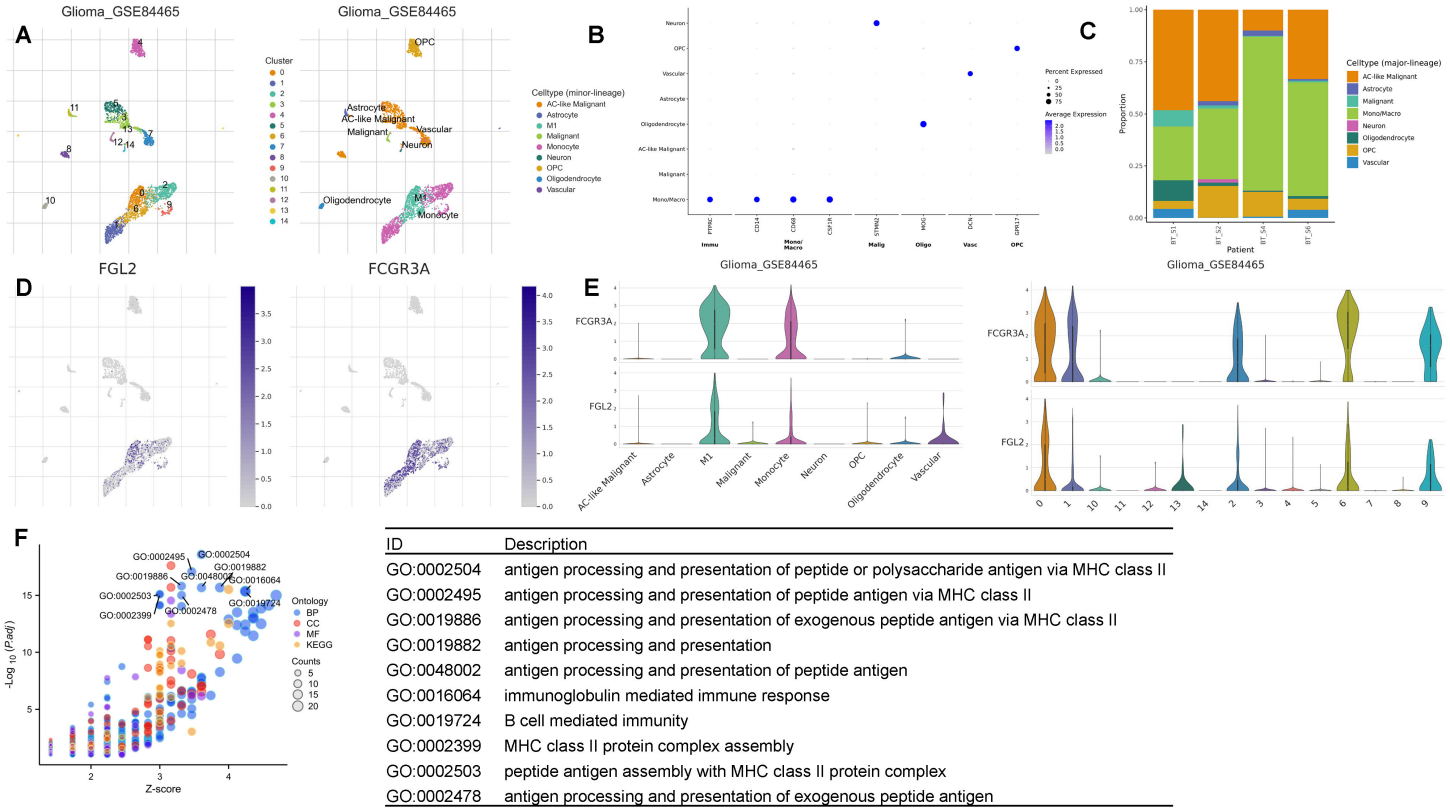
FCGR3A and FGL2 expression in single-cell resolution. **(A, B)** UMAP dimensionality reduction of the single-cell transcriptome dataset GSE84465. **(C)** Cellular proportion. **(D, E)** FCGR3A and FGL2 expression in relative cell type. **(F)** GO enrichment of FCGR3A and FGL2 double positive monocytes.

Given that the expression levels of FCGR3A and FGL2 exhibit similar trends across different subtypes of gliomas and the anatomical localization is the same in tumor, we propose that FCGR3A and FGL2 co express on a type of cell within gliomas. To determine which cell type in glioma with high expression of FGL2 and FCGR3A, we summarized the analysis of the expression levels of FCGR3A and FGL2 in 17 glioma single-cell datasets in **Supplementary Figure 6**, and found that the expression level of FGL2 and FCGR3A are both highest in Mono/Macrophage across all datasets. It can be determined that FCGR3A and FGL2 are co expressed on mononuclear macrophages within the tumor. To further explore the biological role of co-expressed cells, we used GSE84465 single-cell data for analysis, and the results showed that in **Figure 7**. In the GSE84465, there were 9 types of cells, and both monocytes and M1 macrophages expressed FCGR3A highly, while M1 macrophages expressed FGL2 significantly higher than monocytes. Therefore, we hypothesized that FGL2 can assist in the differentiation of monocytes into M1 macrophages. The marker genes of FGL2^+^ macrophages were used for pathway enrichment, and a variety of antigen presentation pathways were activated, which successfully verified the conclusion of bulk RNA-Seq. CD16 has been identified as a marker of non-classical monocytes in the circulation, but it is still unclear whether FGL2 exists during monocyte development and differentiation. To explore whether FGL2 is only induced within tumors, we validated it using single-cell sequencing datasets from healthy human bone marrow and peripheral blood. The results of **Supplementary Figure 7** show that the bone marrow contains immature precursor monocytes and three types of mature monocytes, while CD16^+^non classical monocytes are in the late stage of differentiation trajectory. FCGR3A and FGL2 are gradually upregulated with monocyte development and can be considered as characteristics of mature monocytes. The results of **Supplementary Figure 8** peripheral blood results showed that FGL2 was expressed in almost all mature monocytes/macrophages, but CD16^+^monocytes had the highest level of FGL2 expression. It indicates that FGL2^+^CD16^+^monocytes exist in the peripheral blood under physiological conditions and are recruited through intra-tumoral blood vessels during the occurrence of gliomas.

### The expression of CD16 protein in glioma tissues and macrophages

**Figure 8:**
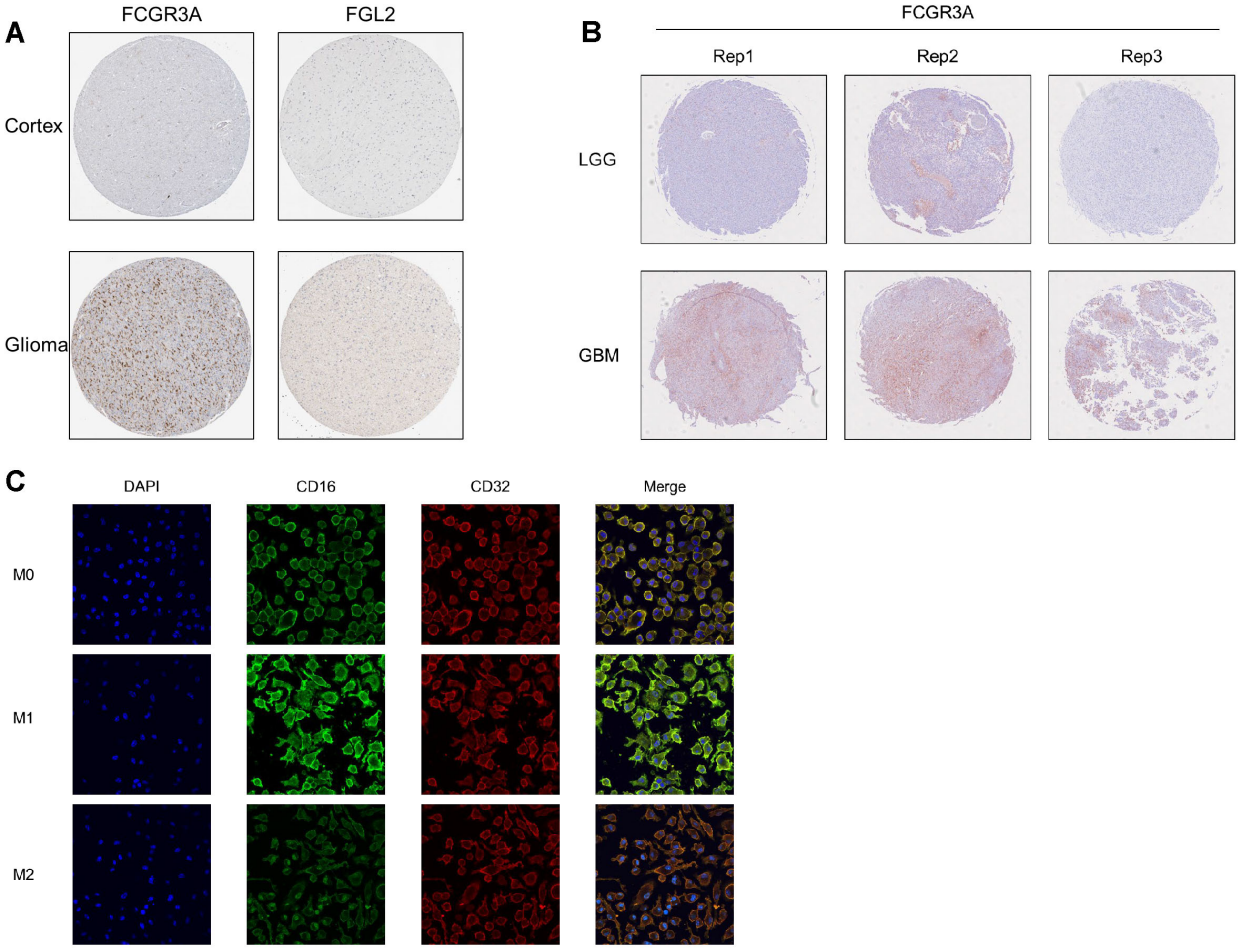
protein level verification of FCGR3A and FGL2 in gliomas. **(A)** HPA database. **(B)** Microarray from Tiantan hospital. **(C)** Induction of differentiation and immunofluorescence detection of human leukemic cell line THP1.

CD16 and FGL2 exhibit their functions at protein level. By searching the protein staining results of CD16 and FGL2 in the HPA database, it shows that normal brain tissue (cortex) does not contain CD16 or FGL2 positive cells, but the number of CD16^+^cells increase significantly in glioma samples, and FGL2 doesn’t show positive staining (**Figure 8A**). However, there are only a few samples in the HPA database, and it does not contain pathological classification. Therefore, using glioma samples collected from Beijing Tiantan Hospital, we created a tissue microarray containing 48 tumor core samples, including 26 LGG samples and 22 GBM samples (**Figure 8B**). Based on this tissue microarray, we conducted pathological examination and histochemical staining for two proteins. The results showed that the cell membrane protein CD16A stained well in most samples, and its positivity rate in LGG was significantly lower than that in GBM. FGL2 protein was not detectable by using two commercially available antibodies in both LGG and GBM samples, consisting with the results of the HPA database. Only 2/12 endometrial cancer and 1/11 melanoma samples are FGL2 positive detected by HPA021011 antibody, and all the samples are negative by HPA026682 antibody in the HPA database. We previously showed that glioma samples were positive for FGL2 by using homemade antibody, indicating that detecting antibody is important for FGL2 staining.

Macrophages are highly heterogeneous and can be activated into different types affected by the local microenvironment, exhibiting different phenotypes and functions. Due to the heterogeneity of macrophages under different tissue environments and physiological and pathological conditions, macrophages are divided into two main types: M1 macrophages and M2 macrophages. By using the human monocyte cell line THP-1 and performing immunofluorescence staining for IgG receptors CD32 and CD16 on the cell membrane after directed induction, we successfully demonstrated that CD16 is positively correlated with the M1 phenotype, rather than CD32 (**Figure 8C**). Further validation is needed for the detection of FGL2 at the protein level. In summary, CD16 protein is highly expressed in M1 macrophages and high-grade glioma.

## Discussion

In recent years, high transcriptional levels of FGL2 have been discovered in various tumors and have predicted an uncertain prognosis among the patients. As a representative of malignancies, glioma, especially GBM, possesses common tumor characteristics including invasion, angiogenesis, epithelial-mesenchymal transition, cancer stem cell-like properties, and metastasis. Previous studies have demonstrated that these malignant features have a positive correlation with high expression of FGL2. Our previous studies believe that FGL2 is synthesized from tumor cells and immune cells. Studies have shown that glioma orthotopic planting model successfully established in mice with systemic knockout of FGL2, but FGL2-KO tumor cells cannot be established in immunocompetent mice. We previously focused on the function of FGL2 in tumors and how it regulates immune response by *in vivo* experiments. This study aims to evaluate the prognostic value of FGL2 transcription expression in glioma, determine the cell types that express FGL2 in human glioma, and to explore how the type of cells affect the generation and progression of glioma. In addition, trying to detect FGL2 *in situ* on pathological sections is also one of our goals, although the results are not ideal.

Due to the small sample size and uncertainty of sample quality, we abandoned the traditional research methods and turned to data rich online tools including GEPIA, and CGGA, etc. We can acquire a more authentic and comprehensive perspective using different online databases, which provide more information on tumors, both supplementing and verifying each other. The data of patients and gliomas are mainly from TCGA and CGGA; the former involves the Western region while CGGA only includes Chinese patients. In consideration of the rarity of WHO I glioma, we only regard LGG as WHO II and III gliomas.

Our results show that in mRNA level, FGL2 is only expressed in CD16^+^ myeloid cells in gliomas, a subpopulation mainly responsible for phagocytosis, antigen processing, presentation, and activation of T-cells. This type of myeloid cells is mainly formed by the migration of non-classical monocytes in peripheral blood. FGL2 can assist in the activation of CD16^+^ cells, which increases with the degree of infiltration. FCGR3A^+^monocytes, by co-expressing FGL2 on the extracellular surface, activates a T cell activation pathway, which are involved in cytokine producing, MHC complex binding and cell migration. As functional integrity, the FCGR3A-FGL2 synergistic effect is supposed to not only plays a crucial role in mediating pro-inflammation during cancer invasion and metastasis but also participates into multiple stages of tumor proliferation.

FCGR3A and FGL2 transcriptional expressions are found significantly higher in glioma compared with normal brain. The FGL2 expression predicts poor prognosis only in LGG, and is not associated with compromised prognosis in High Grade Glioma. In GBM and part of recurrent gliomas, the prognostic value is questionable. There might be two possible explanations. On the one hand, high heterogeneity of malignant glioma leads to the diversity of OS; on the other hand, the survival period of GBM and recurrent tumor patients is relatively too short. The results of Cox regression analysis further demonstrate the independent prognostic significance of FCGR3A and FGL2 in glioma. Interestingly, the two genes are similarly in the shape of the Kaplan-Mier plots, especially in the CGGA database. High Pearson correlation efficiency between FCGR3A and FGL2 indicates that the two genes share the same prognosis significance and could be integrated into one prognostic prediction model.

In view of the high incidence of genetic mutations in glioma, cBioPortal is used to evaluate the gene alteration frequency and whether the alteration affects overall survival. As shown in **Figure 1**, the gene alteration frequencies of 2 genes are negligible, without impact on overall survival. It means FCGR3A and FGL2 transcriptional expression are less affected by genetic mutations which usually cause phenotypic changes.

Extracellular matrix (ECM) breakdown is an important step for cell invasion and metastasis, and FCGR3A and FGL2 may play a key role in this process (22, 23). Growing evidences indicate that CD16+ cells secrete more inflammatory cytokines, and interleukin and tumor necrosis factor can induce degradation of the extracellular matrix. For example, interleukin-1β (IL-1β) and tumor necrosis factor α (TNF-α) can stimulate the production of matrix metalloproteinases (MMPs), enzymes that can degrade various components of the extracellular matrix, such as collagen and proteoglycans.

FCGR3A and FGL2 co-expression network is constructed to further analyze their role in cell function and signaling pathway. They share a lot of similarities in the gene set enrichment analysis. They both had a close relationship with adaptive immune cells activation like T cell activation process. The FGL2-deficient mice failed to develop tumor, suggesting that FGL2-mediated antigen presentation does not seem to successfully activate the anti-tumor effect of T cells. It is also indicated in the previous research that FGL2 mediates regulatory T cells and MDSCs suppressor function through inducing ROS production and promotes the activation of the XBP1 pathway and cholesterol synthesis in sepsis, which characterized by immune paralysis. It is also reported that FGL2 plays a role in lymphocyte migration.

At the same time, the close relationship between the FCGR3A and FGL2 expression and immune cells might be a key factor in the development of cirrhosis of the liver. Except for some inflammation-related signaling pathways, several fibrosis-promoting pathways have a relationship with FCGR3A and FGL2 co-expression networks (24, 25). The CD14^+^CD16^+^ monocyte subpopulation is an important inflammatory cell population involved in tissue fibrosis. In acute respiratory distress syndrome (ARDS) caused by COVID-19, CD16-expressing monocyte-derived macrophages accumulate and exhibit a fibrosis-promoting transcriptional phenotype (26, 27). The number of CD16^+^ cells is positively correlated with liver damage and the progression of liver fibrosis in patients with hepatitis B. For patients with pancreatic cancer, CD16^+^ monocytes can activate pancreatic stellate cells and promote the formation of local fibrosis in pancreatic cancer. Similarly, FGL2 has been reported to promote the progression of liver fibrosis by regulating the polarization state of macrophages (28, 29). Compared to wild mice, liver injury was significantly reduced in the hepatitis model of Fgl2-deficient mice. In kidney fibrosis models, the expression of FGL2 increases significantly and is closely related to the pathological changes of renal fibrosis.

Fibrosis plays a crucial role in the growth and development of gliomas. Gliomas are infiltrating tumors that grow without distinct boundaries from normal brain tissue, causing significant damage to surrounding brain structures. As a result, glioma tissue often contains abundant fibrous tissue formed by extracellular matrix (ECM) remodeling and excessive collagen deposition (30). Fibrosis provides structural support for glioma cells, contributing to the stability and growth of the tumor tissue. The fibrous network it forms also supports the proliferation and expansion of glioma cells. Fibrotic glioma tissue reduces the infiltration and function of immune cells, creating an immunosuppressive microenvironment that aids in the immune evasion of glioma cells. Additionally, fibrosis hinders the penetration and diffusion of therapeutic drugs, thereby reducing treatment efficacy. This is because the fibrous tissue structure makes it difficult for drugs to penetrate the interior of the tumor.

There are some limitations in this study. First, the main content of this study focuses on retrospective analysis derived from TCGA and CGGA and GEO databases, our conclusions need to be validated by more *in vivo* or *in vitro* experiments and prospective clinical studies. In the future, we will knock out FGL2 on CD16^+^ cells to see if polarization function is affected. CCR5 will be blocked in mice to see if it could affect the infiltration of monocytes into nerve tissue. In addition, the mechanism of CD16 reduction after tumor invasion also needs to be investigated, and the role of cis-binding of CD16 to FGL2 on monocytic phagocytosis needs to be further explored.

In conclusion, our current study indicated that overexpression of FCGR3A and FGL2 is associated with poor prognosis in primary and recurrent glioma patients, especially in LGG. Overexpression of FCGR3A and FGL2 is regarded as independent prognostic factors for shorter OS of glioma patients through Cox regression analysis. Moreover, Gene co-expression network analysis enlightens us that abnormal adaptive immune activation and specific CD16-related Immune paralysis blocking by targeting FCGR3A and FGL2 might be a new idea for treating glioma.

## Data Availability

Apart from the publicly available online databases (as accessed through the URLs provided in the Methods section), the single-cell sequencing data presented in this study are derived from a public dataset in the GEO repository, with the accession number GSE84465 (https://www.ncbi.nlm.nih.gov/geo/query/acc.cgi?acc=GSE84465).

## Supplementary material

Supplementary Figure 1 Protein expression and epigenetic modification of FCGR3A and FGL2. **(A, B)** Protein expression of FCGR3A and FGL2 in gliomas and normal brain tissues(CPTAC). **(C, D)** Protein expression of FCGR3A and FGL2 in pan-cancers (CPTAC). **(E, F)** Promoter region methylation level of FCGR3A and FGL2 in gliomas and normal brain tissues (TCGA). **(F, G)** Transcription factors and post-transcriptional modifications of FCGR3A and FGL2 in gliomas(TCGA). Note: As the LGG samples in the TCGA database do not contain normal tissues, only the results of GBM are displayed here.

Supplementary Figure 2 Expression difference of FCGR3A and FGL2. **(A)** Transcriptional expression of FCGR3A and FGL2 in normal tissues (GTEx). **(B, C)** Transcriptional expression of FCGR3A and FGL2 in Pan-cancer (TCGA). **(D-H)** Expression difference of FCGR3A and FGL2 in glioma sub-groups. *p < 0.01, * *p < 0.001, *p < 0.0001.

Supplementary Figure 3 Prognostic effect of FCGR3A in glioma sub-groups.

Supplementary Figure 4 Prognostic effect of FGL2 in glioma sub-groups.

Supplementary Figure 5 Construction of prognostic model by using FCGR3A and FGL2. **(A-C)** Prediction effect of OS of FCGR3A and FGL2 in gliomas. **(D, E)** Construction of prognostic model.

Supplementary Figure 6 Summary of FCGR3A and FGL2 expression in multiple single-cell datasets.

Supplementary Figure 7 Developmental trajectory of FCGR3A and FGL2 in monocytes.

Supplementary Figure 8 FGL2+CD16+monocytes present in peripheral blood of healthy controls. **(A-C)** Prediction effect of OS of FCGR3A and FGL2 in gliomas. **(D, E)** Construction of prognostic model.

## References

[1] Guo Z Su Z Wei Y Zhang X Hong X. Pyroptosis in glioma: Current management and future application. Immunol Rev. (2024) 321:152–68. doi: 10.1111/imr.13294 38063042

[2] Gusyatiner O Hegi ME. Glioma epigenetics: From subclassification to novel treatment options. Semin Cancer Biol. (2018) 51:50–8. doi: 10.1016/j.semcancer.2017.11.010 29170066

[3] Malta TM de Souza CF Sabedot TS Silva TC Mosella MS Kalkanis SN. Glioma CpG island methylator phenotype (G-CIMP): biological and clinical implications. Neuro Oncol. (2018) 20:608–20. doi: 10.1093/neuonc/nox183 PMC5892155 29036500

[4] Yasinjan F Xing Y Geng H Guo R Yang L Liu Z. Immunotherapy: a promising approach for glioma treatment. Front Immunol. (2023) 14:1255611. doi: 10.3389/fimmu.2023.1255611 37744349 PMC10512462

[5] Chen R Smith-Cohn M Cohen AL Colman H. Glioma subclassifications and their clinical significance. Neurotherapeutics. (2017) 14:284–97. doi: 10.1007/s13311-017-0519-x PMC5398991 28281173

[6] Yin Q Song D Chen J Ning G Wang W Wang S. The CD14(++) CD16(+) monocyte subset is expanded and controls Th1 cell development in Graves’ disease. Clin Immunol. (2022) 245:109160. doi: 10.1016/j.clim.2022.109160 36270470

[7] Calabrese DR Chong T Singer JP Rajalingam R Hays SR Kukreja J. CD16(+) natural killer cells in bronchoalveolar lavage are associated with antibody-mediated rejection and chronic lung allograft dysfunction. Am J Transplant. (2023) 23:37–44. doi: 10.1016/j.ajt.2022.10.006 36695619 PMC10018437

[8] Wacleche VS Cattin A Goulet JP Gauchat D Gosselin A Cleret-Buhot A. CD16(+) monocytes give rise to CD103(+) RALDH2 (+) TCF4(+) dendritic cells with unique transcriptional and immunological features. Blood Adv. (2018) 2:2862–78. doi: 10.1182/bloodadvances.2018020123 PMC6234376 30381402

[9] Janssen E Alosaimi MF Alazami AM Alsuliman A Alaiya A Al-Saud B. A homozygous truncating mutation of FGL2 is associated with immune dysregulation. J Allergy Clin Immunol. (2023) 151:572–78. doi: 10.1016/j.jaci.2022.10.006 36243222

[10] Huang L Zhan D Xing Y Yan Y Li Q Zhang J. FGL2 deficiency alleviates maternal inflammation-induced blood-brain barrier damage by blocking PI3K/NF-kappaB mediated endothelial oxidative stress. Front Immunol. (2023) 14:1157027. doi: 10.3389/fimmu.2023.1157027 37051251 PMC10083319

[11] Chen JY Wang CM Chang SW Cheng CH Wu YJ Lin JC. Association of FCGR3A and FCGR3B copy number variations with systemic lupus erythematosus and rheumatoid arthritis in Taiwanese patients. Arthritis Rheumatol. (2014) 66:3113–21. doi: 10.1002/art.38813 PMC4232894 25154742

[12] Cooper N Stasi R Cunningham-Rundles S Cesarman E McFarland JG Bussel JB. Platelet-associated antibodies, cellular immunity and FCGR3a genotype influence the response to rituximab in immune thrombocytopenia. Br J Haematol. (2012) 158:539–47. doi: 10.1111/j.1365-2141.2012.09184.x 22775462

[13] Alizadeh ZH Yeoh WJ Arif M Lomora M Banz Y Riether C. Natural killer cell-mimic nanoparticles can actively target and kill acute myeloid leukemia cells. Biomaterials. (2023) 298:122126. doi: 10.1016/j.biomaterials.2023.122126 37094524

[14] Zhao Q Hu J Kong L Jiang S Tian X Wang J. FGL2-targeting T cells exhibit antitumor effects on glioblastoma and recruit tumor-specific brain-resident memory T cells. Nat Commun. (2023) 14:735. doi: 10.1038/s41467-023-36430-2 36759517 PMC9911733

[15] Wu L Liu X Lei J Zhang N Zhao H Zhang J. Fibrinogen-like protein 2 promotes tumor immune suppression by regulating cholesterol metabolism in myeloid-derived suppressor cells. J Immunother Cancer. (2023) 11(12):e008081. doi: 10.1136/jitc-2023-008081 38056898 PMC10711877

[16] Yu J Li J Shen J Du F Wu X Li M. The role of Fibrinogen-like proteins in Cancer. Int J Biol Sci. (2021) 17:1079–87. doi: 10.7150/ijbs.56748 PMC8040309 33867830

[17] Li Z Zhou B Zhu X Yang F Jin K Dai J. Differentiation-related genes in tumor-associated macrophages as potential prognostic biomarkers in non-small cell lung cancer. Front Immunol. (2023) 14:1123840. doi: 10.3389/fimmu.2023.1123840 36969247 PMC10033599

[18] Feng Y Guo C Wang H Zhao L Wang W Wang T. Fibrinogen-like protein 2 (FGL2) is a novel biomarker for clinical prediction of human breast cancer. Med Sci Monit. (2020) 26:e923531. doi: 10.12659/MSM.923531 32716910 PMC7409386

[19] Galpin K Rodriguez GM Maranda V Cook DP Macdonald E Murshed H. FGL2 promotes tumour growth and attenuates infiltration of activated immune cells in melanoma and ovarian cancer models. Sci Rep. (2024) 14:787. doi: 10.1038/s41598-024-51217-1 38191799 PMC10774293

[20] Yan J Zhao Q Wang J Tian X Wang J Xia X. FGL2-wired macrophages secrete CXCL7 to regulate the stem-like functionality of glioma cells. Cancer Lett. (2021) 506:83–94. doi: 10.1016/j.canlet.2021.02.021 33676940 PMC8009861

[21] Yan J Zhao Q Gabrusiewicz K Kong LY Xia X Wang J. FGL2 promotes tumor progression in the CNS by suppressing CD103(+) dendritic cell differentiation. Nat Commun. (2019) 10:448. doi: 10.1038/s41467-018-08271-x 30683885 PMC6347641

[22] Kiyokawa J Kawamura Y Ghouse SM Acar S Barcin E Martinez-Quintanilla J. Modification of extracellular matrix enhances oncolytic adenovirus immunotherapy in glioblastoma. Clin Cancer Res. (2021) 27:889–902. doi: 10.1158/1078-0432.CCR-20-2400 33257429 PMC7854507

[23] Hancock WW Szaba FM Berggren KN Parent MA Mullarky IK Pearl J. Intact type 1 immunity and immune-associated coagulative responses in mice lacking IFN gamma-inducible fibrinogen-like protein 2. Proc Natl Acad Sci U.S.A. (2004) 101:3005–10. doi: 10.1073/pnas.0308369101 PMC365735 14976252

[24] Foerster K Helmy A Zhu Y Khattar R Adeyi OA Wong KM. The novel immunoregulatory molecule FGL2: a potential biomarker for severity of chronic hepatitis C virus infection. J Hepatol. (2010) 53:608–15. doi: 10.1016/j.jhep.2010.04.020 20615566

[25] Angelini G Panunzi S Castagneto-Gissey L Pellicano F De Gaetano A Pompili M. Accurate liquid biopsy for the diagnosis of non-alcoholic steatohepatitis and liver fibrosis. Gut. (2023) 72:392–403. doi: 10.1136/gutjnl-2022-327498 35820779 PMC9872242

[26] Manohar M Jones EK Rubin S Subrahmanyam PB Swaminathan G Mikhail D. Novel circulating and tissue monocytes as well as macrophages in pancreatitis and recovery. Gastroenterology. (2021) 161:2014–29. doi: 10.1053/j.gastro.2021.08.033 PMC8796698 34450180

[27] Hu J Wang H Li X Liu Y Mi Y Kong H. Fibrinogen-like protein 2 aggravates nonalcoholic steatohepatitis via interaction with TLR4, eliciting inflammation in macrophages and inducing hepatic lipid metabolism disorder. Theranostics. (2020) 10:9702–20. doi: 10.7150/thno.44297 PMC7449923 32863955

[28] Stephenson E Reynolds G Botting RA Calero-Nieto FJ Morgan MD Tuong ZK. Single-cell multi-omics analysis of the immune response in COVID-19. Nat Med. (2021) 27:904–16. doi: 10.1038/s41591-021-01329-2 PMC8121667 33879890

[29] Zhang J Gao C Zhu Z Li D Qu L Xue Q. New findings on CD16(bright)CD62L(dim) neutrophil subtypes in sepsis-associated ARDS: an observational clinical study. Front Immunol. (2024) 15:1331050. doi: 10.3389/fimmu.2024.1331050 38605959 PMC11007181

[30] Zhong C Tao B Tang F Yang X Peng T You J. Remodeling cancer stemness by collagen/fibronectin via the AKT and CDC42 signaling pathway crosstalk in glioma. Theranostics. (2021) 11:1991–2005. doi: 10.7150/thno.50613 33408794 PMC7778591

