# Supplementary figures and images for: Prognostic significance and gene co-expression network of CD16A and FGL2 in gliomas
Source: Front Oncol. 2024 Nov 19;14:1447113. doi: 10.3389/fonc.2024.1447113 (PMC11611834; doi:10.3389/fonc.2024.1447113)

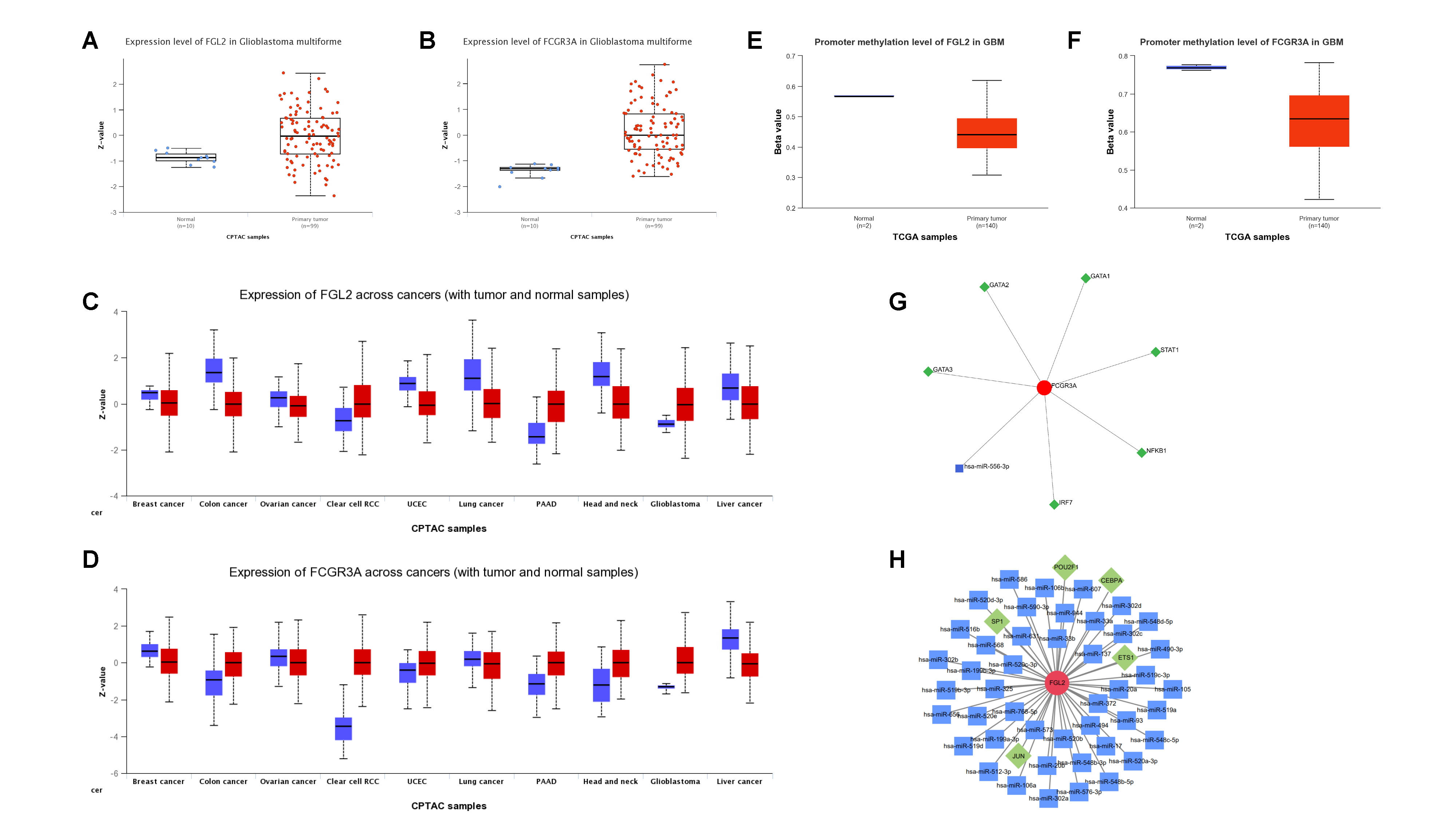

Supplement: Supplementary Figure 1 — Protein expression and epigenetic modification of FCGR3A and FGL2. (A, B) Protein expression of FCGR3A and FGL2 in gliomas and normal brain tissues(CPTAC). (C, D) Protein expression of FCGR3A and FGL2 in pan-cancers (CPTAC). (E, F) Promoter region methylation level of FCGR3A and FGL2 in gliomas and normal brain tissues (TCGA). (F, G) Transcription factors and post-transcriptional modifications of FCGR3A and FGL2 in gliomas(TCGA). Note: As the LGG samples in the TCGA database do not contain normal tissues, only the results of GBM are displayed here. [file Image1.tiff]

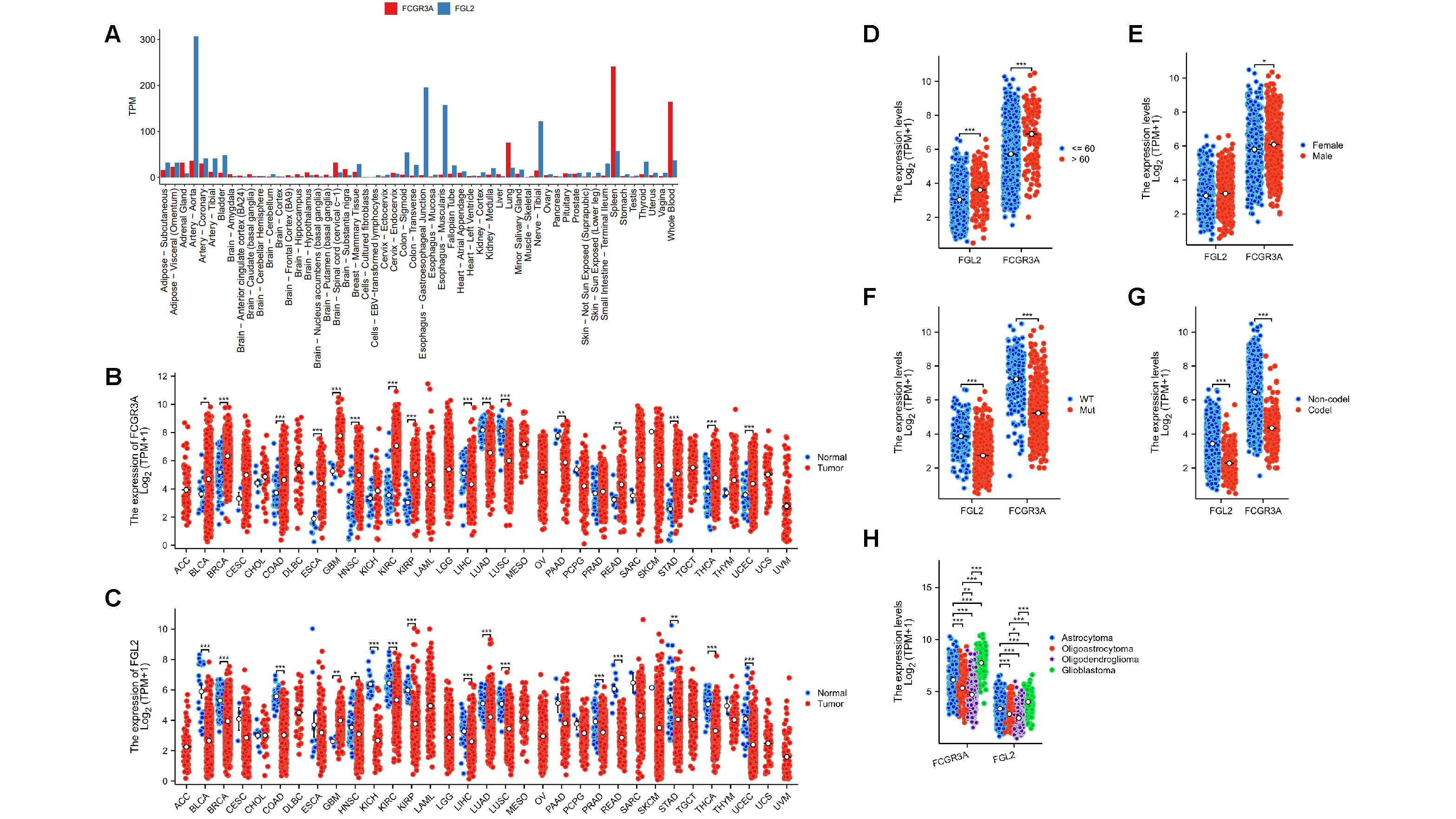

Supplement: Supplementary Figure 2 — Expression difference of FCGR3A and FGL2. (A) Transcriptional expression of FCGR3A and FGL2 in normal tissues (GTEx). (B, C) Transcriptional expression of FCGR3A and FGL2 in Pan-cancer (TCGA). (D-H) Expression difference of FCGR3A and FGL2 in glioma sub-groups. *p < 0.01, * *p < 0.001, *p < 0.0001. [file Image2.tiff]

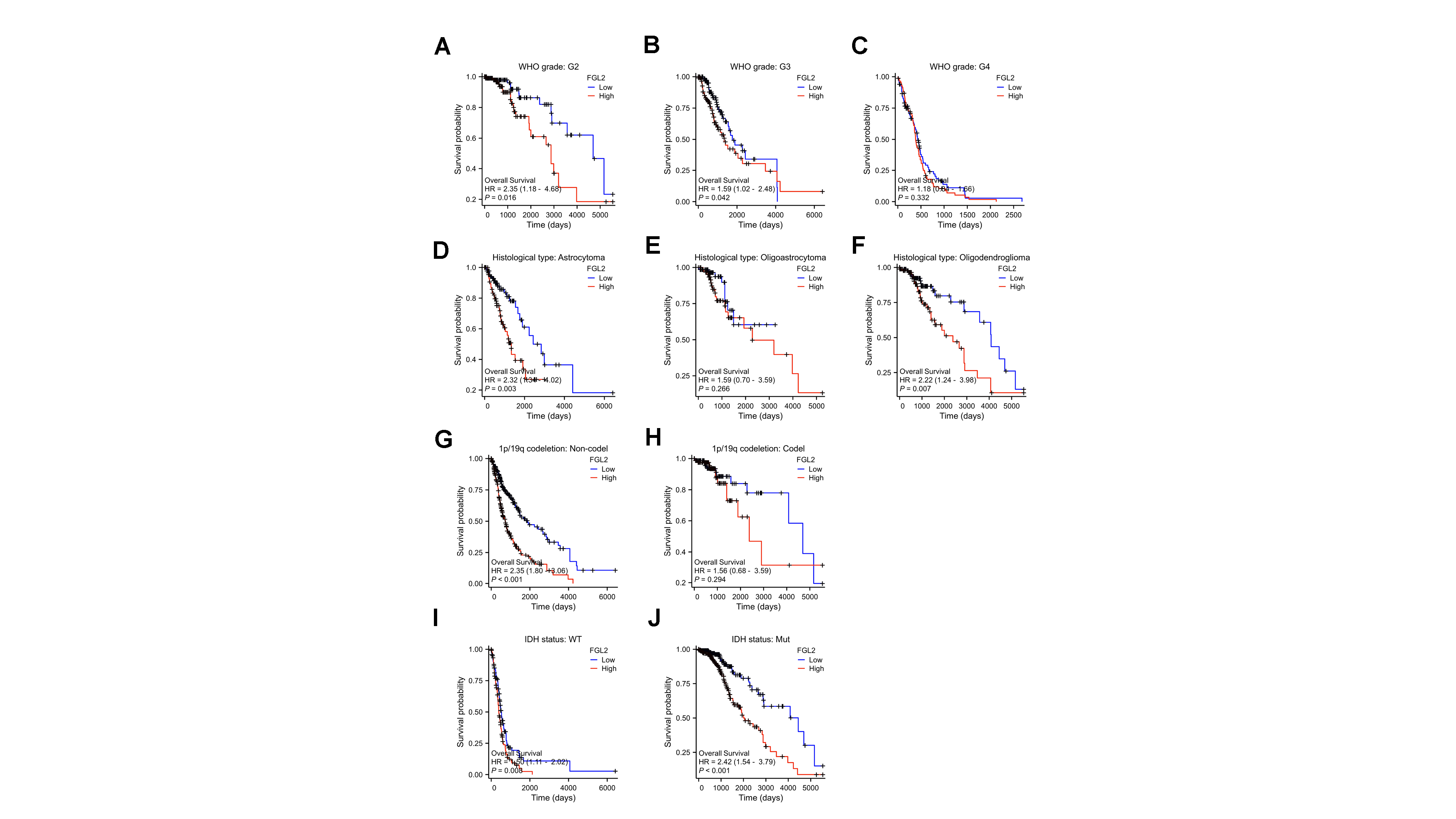

Supplement: Supplementary Figure 3 — Prognostic effect of FCGR3A in glioma sub-groups. [file Image3.tiff]

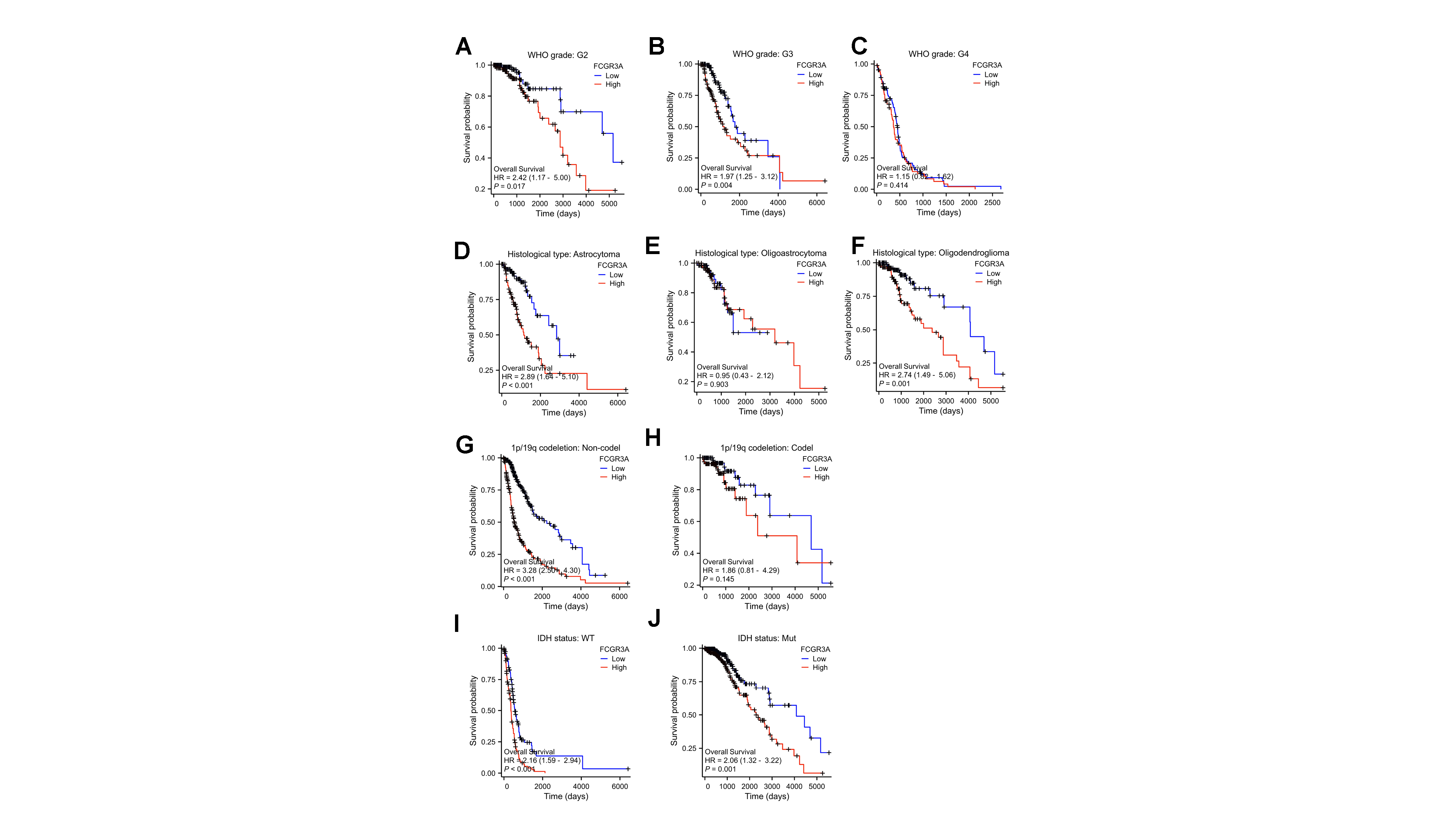

Supplement: Supplementary Figure 4 — Prognostic effect of FGL2 in glioma sub-groups. [file Image4.tiff]

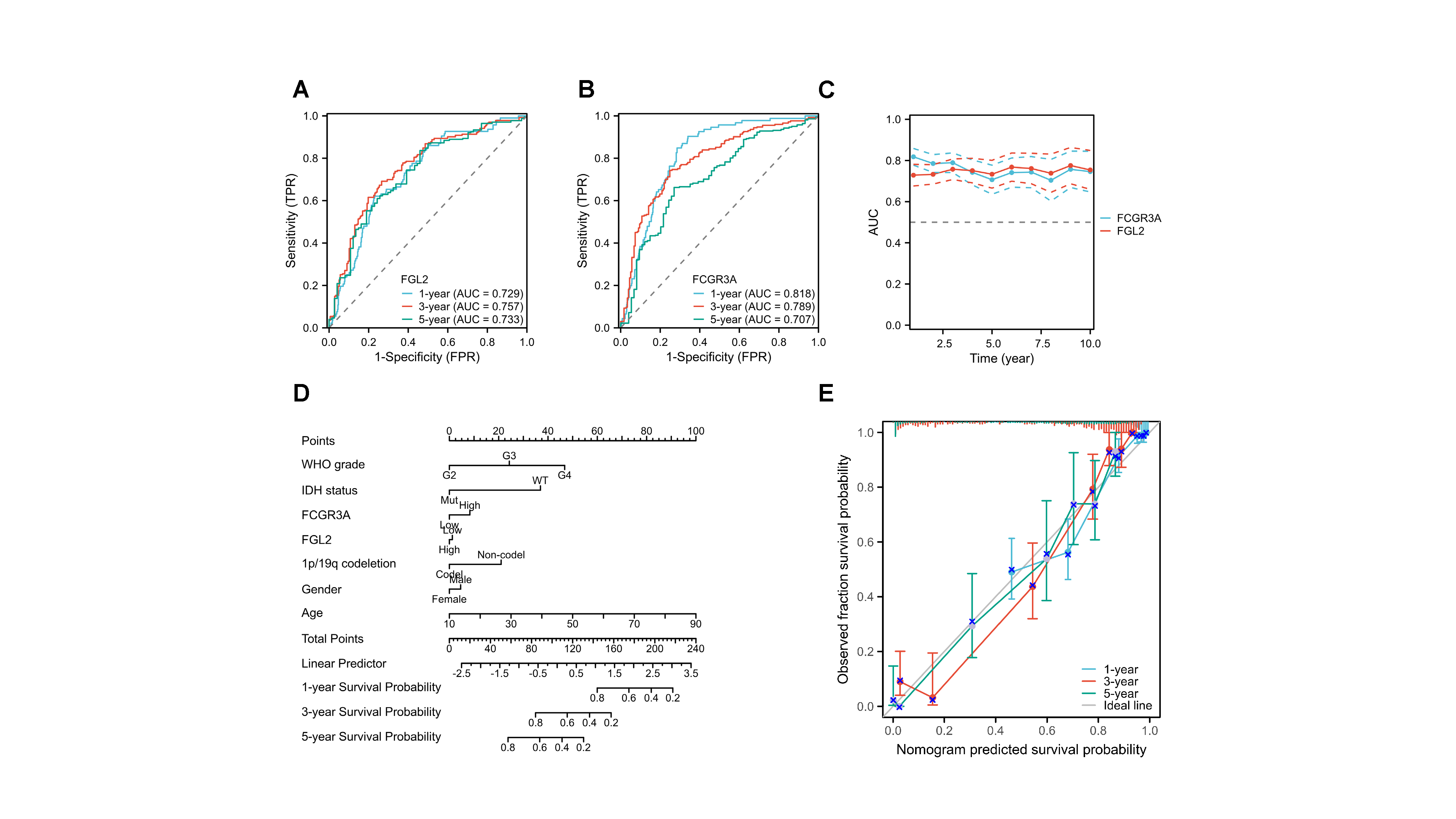

Supplement: Supplementary Figure 5 — Construction of prognostic model by using FCGR3A and FGL2. (A-C) Prediction effect of OS of FCGR3A and FGL2 in gliomas. (D, E) Construction of prognostic model. [file Image5.tiff]

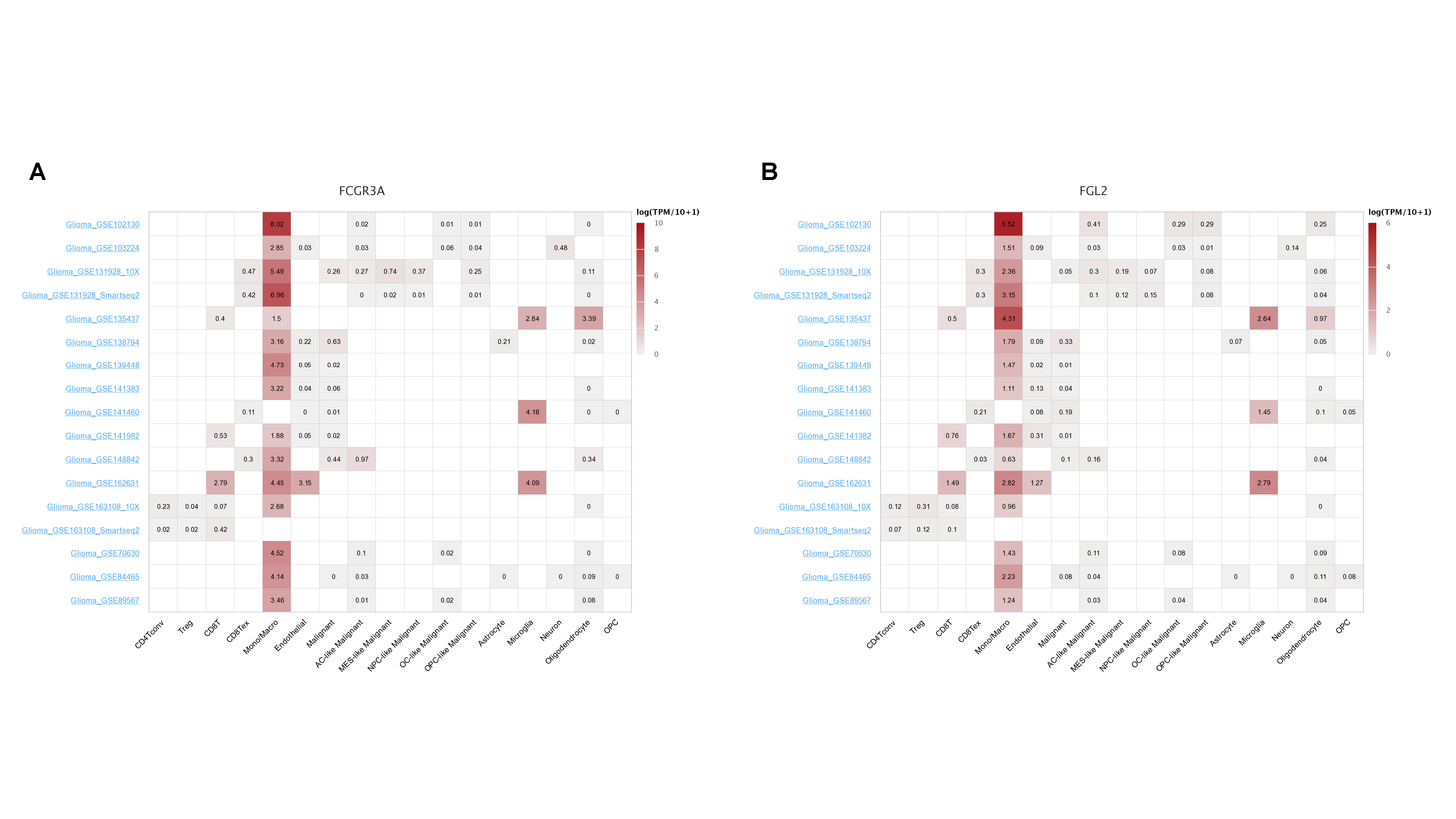

Supplement: Supplementary Figure 6 — Summary of FCGR3A and FGL2 expression in multiple single-cell datasets. [file Image6.tiff]

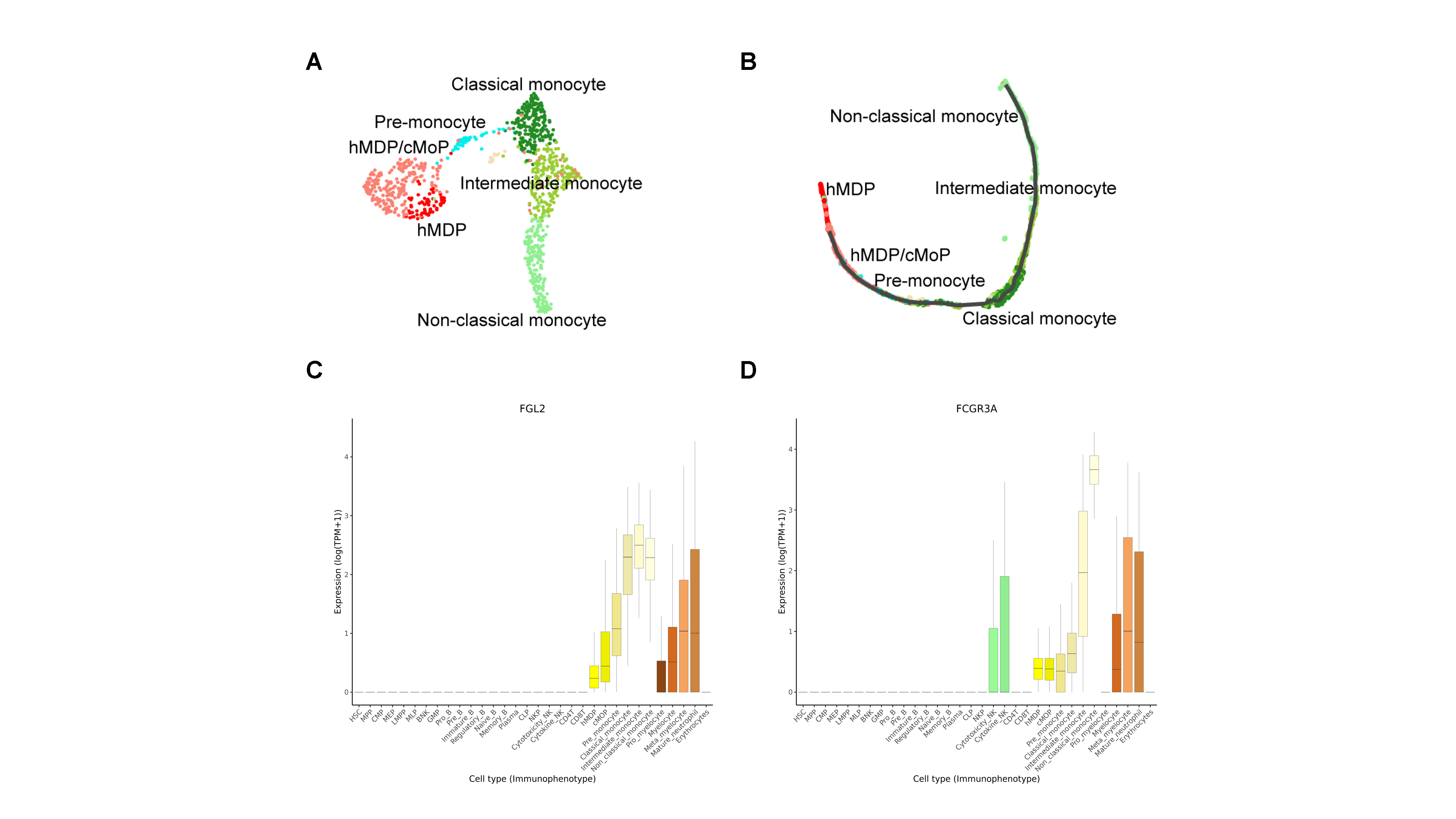

Supplement: Supplementary Figure 7 — Developmental trajectory of FCGR3A and FGL2 in monocytes. [file Image7.tiff]

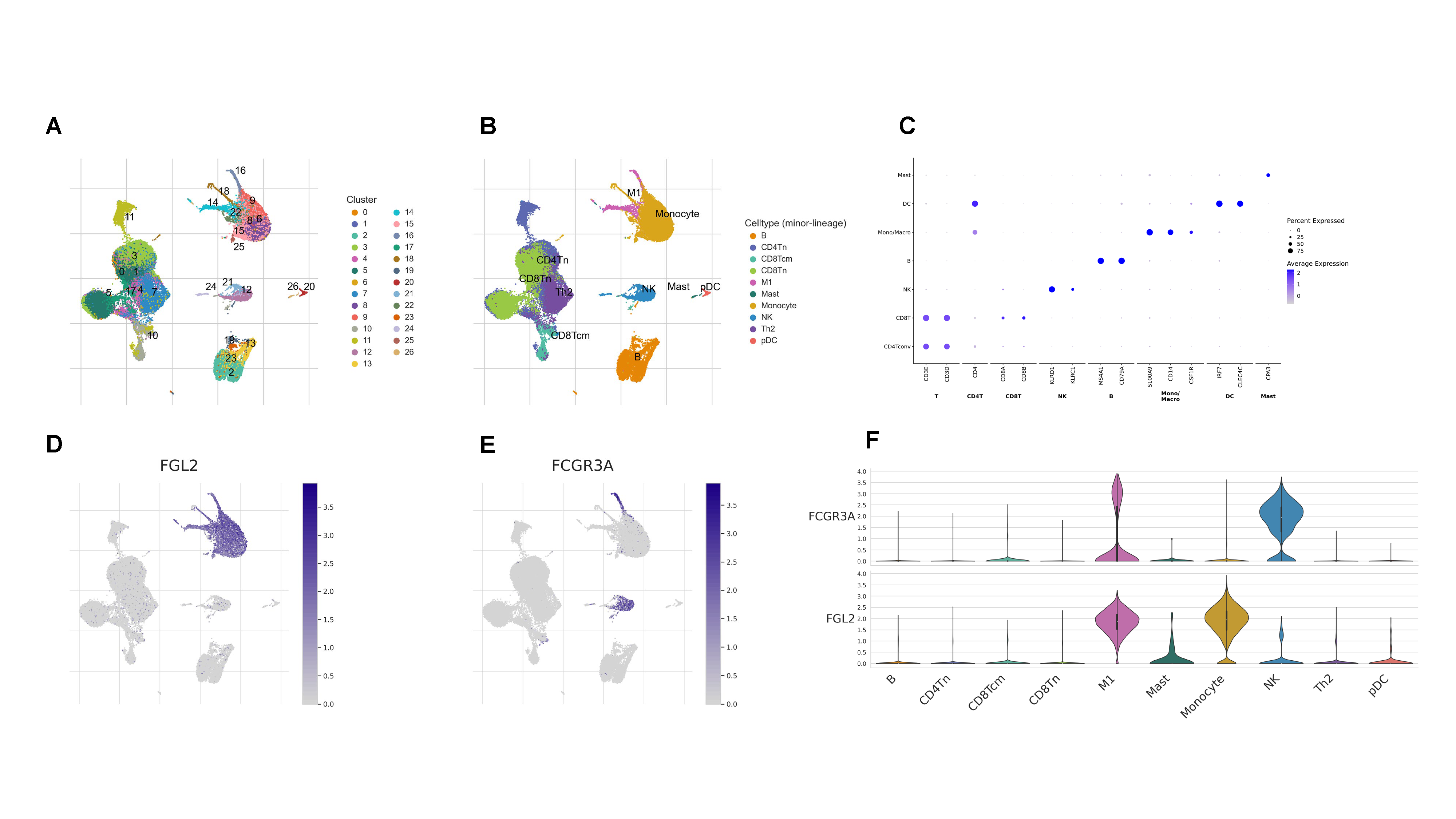

Supplement: Supplementary Figure 8 — FGL2+CD16+monocytes present in peripheral blood of healthy controls. (A-C) Prediction effect of OS of FCGR3A and FGL2 in gliomas. (D, E) Construction of prognostic model. [file Image8.tiff]
